# Thermoelectric energy harvesting for personalized healthcare

**DOI:** 10.1002/SMMD.20220016

**Published:** 2022-12-22

**Authors:** Wei Gao, Yang Wang, Feili Lai

**Affiliations:** ^1^ John A. Paulson School of Engineering and Applied Sciences Harvard University Cambridge Massachusetts USA; ^2^ Department of Chemistry KU Leuven Leuven Belgium; ^3^ Department of Molecular Spectroscopy Max Planck Institute for Polymer Research Mainz Germany

**Keywords:** body heat, energy conversion, implantability, thermoelectrics, wearability

## Abstract

In recent decades, there has been increased research interest in miniaturizing and decentralizing diagnostic platforms to enable continuous personalized healthcare and free patients from long‐term hospitalization. However, the lack of reliable and portable power supplies has limited the working time of the personalized healthcare platform. Compared with the current power supplies (e.g., batteries and supercapacitors) that require manual intervention, thermoelectric devices promise to continuously harvest waste heat from the human body to satisfy the energy consumption of personalized healthcare platforms. Herein, this review discusses thermoelectric energy harvesting for personalized healthcare. It begins with the fundamental concepts of different thermoelectric materials, including electron thermoelectric generators (TEGs), ionic thermogalvanic cells (TGCs), and ionic thermoelectric capacitors (TECs). Then, the wearable and implantable applications of thermoelectric devices are presented. Finally, future directions of next‐generation thermoelectric devices for personalized healthcare are discussed. It is hoped that developing high‐performance thermoelectric devices will change the landscape of personalized healthcare in the future.

## Graphical abstract

The power supplies have limited the working time of the portable personalized healthcare platform due to their periodic recharging. Thermoelectric devices can continuously harvest body heat to satisfy the energy consumption of personalized healthcare platforms. This review discusses the fundamental concepts and medical applications of thermoelectric materials and further provides a perspective for designing next‐generation thermoelectric devices.

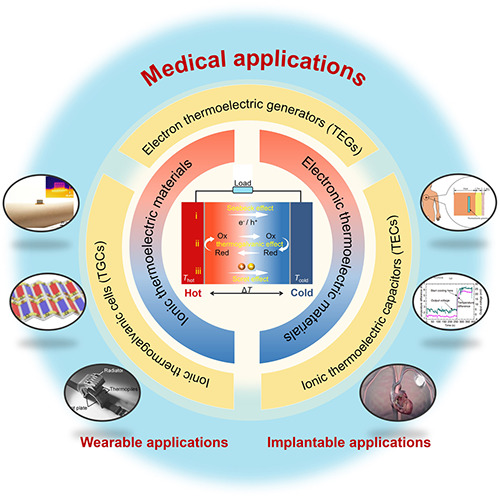

1

**Key points**

- Fundamental concepts of electronic and ionic thermoelectric materials are examined.
- The wearable and implantable applications of thermoelectric devices are discussed.
- Future directions of next‐generation thermoelectric devices for personalized healthcare are presented.

## 2 INTRODUCTION

With the increasing demand of the global population for accessible, affordable, and quality biomedical platforms, miniaturized and decentralized personalized healthcare access has attracted increasing attention due to the potent monitoring of vital signs and remote diagnostics outside the clinical environment.^1^, ^2^ Wearable and implantable platforms that could continuously collect health information from human bodies are attractive clinical tools for personalized diagnostics and therapy.^3^, ^4^ Personalized healthcare can help free patients from bulky biomedical platforms and long‐term hospitalization.^5^ However, the lack of reliable and portable power supplies inevitably impedes the development of wearable and implantable healthcare devices. The challenge is even more pronounced for implantable devices, which require a long working time to continuously monitor body situations or provide auxiliary functions. Therefore, low‐cost, maintenance‐free, and high‐performance power supplies with a continuous working mode are demanded for personalized healthcare.

**FIGURE 1:**
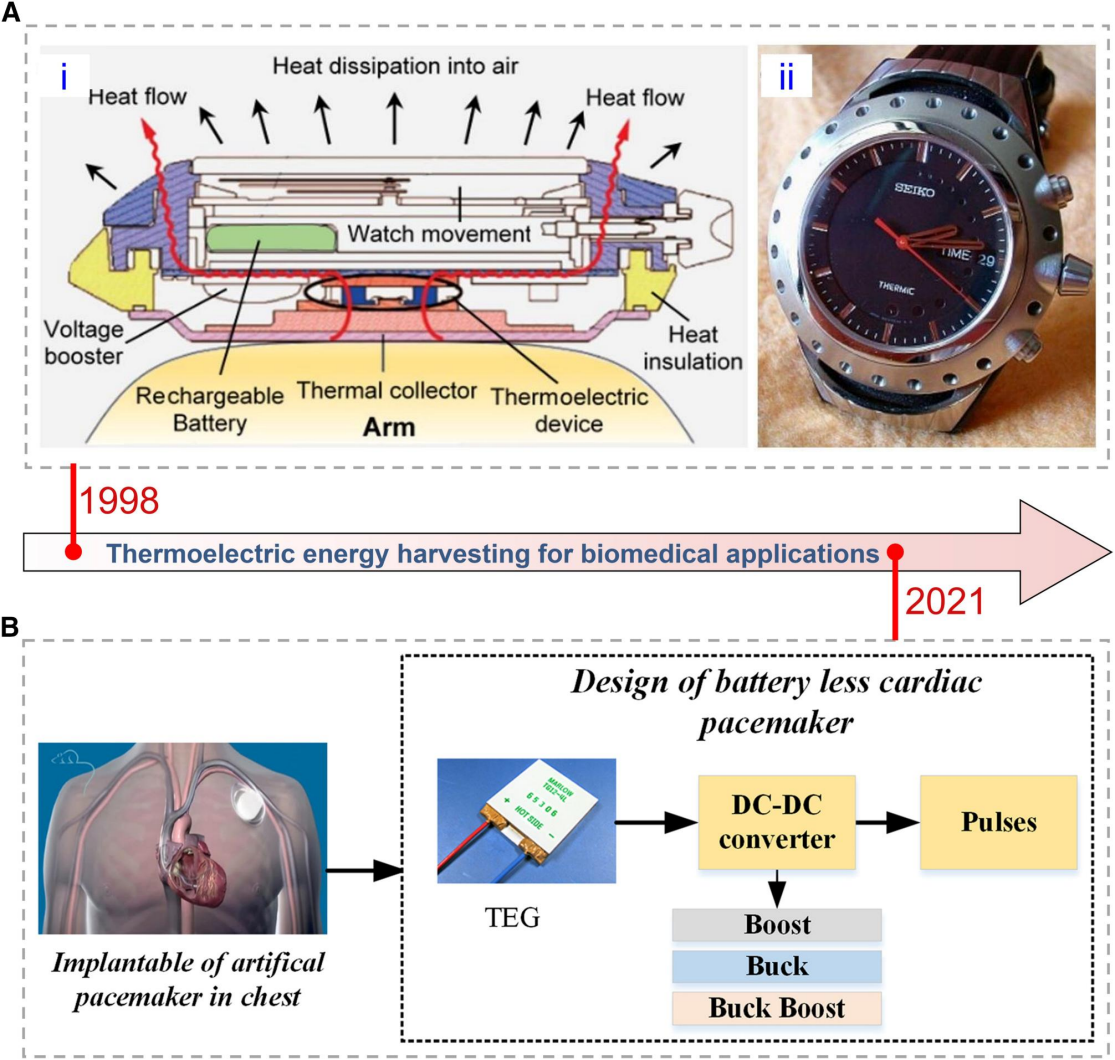
The development of thermoelectrics in wearable and implantable applications. (A) The first wearable thermoelectric device with a radiator.^22^ (Reprinted with permission. Copyright 2020, Elsevier). (B) The implantable thermoelectric device for the pacemaker.^23^ (Reprinted with permission. Copyright 2021, John Wiley and Sons).

Micro batteries^6^, ^7^ and supercapacitors^8^, ^9^ have been widely studied for power supplies; however, the requirements for periodic recharging and replacement limit their long‐term application in healthcare and remote diagnostics. Other portable power supplies, such as solar cells,^10^ piezoelectric,^11^ and triboelectric generators,^12^ only provide energy periodically depending on the working environment. In particular, these power supplies require periodic and complicated manual inspections to support implanted devices working in the human body.^13^, ^14^ Fortunately, the human body can continuously dissipate thermal energy about 20 mW cm^−2^,^15^ promising to be harvested by thermoelectrics to satisfy the energy consumption of personalized healthcare platforms (e.g., biosensors <50 µW, communication transmission <1.5 mW).^16^, ^17^ Recent innovations of thermoelectric devices in the application of wearable and implantable personalized healthcare have made this continuous energy harvesting approach a promising candidate for miniaturized and decentralized biomedical diagnostic platforms (Figure 1).^18^, ^19^, ^20^, ^21^, ^22^, ^23^

This review discusses thermoelectric energy harvesting for personalized healthcare. It first introduces fundamental concepts of different thermoelectric materials, including electron thermoelectric generators (TEGs), ionic thermogalvanic cells (TGCs), and ionic thermoelectric capacitors (TECs). Then, it presents wearable and implantable applications of thermoelectric devices. Finally, the challenges and opportunities of the next‐generation thermoelectric devices for personalized healthcare are discussed.

## 3 FUNDAMENTALS OF THE THERMOELECTRICS

**FIGURE 2:**
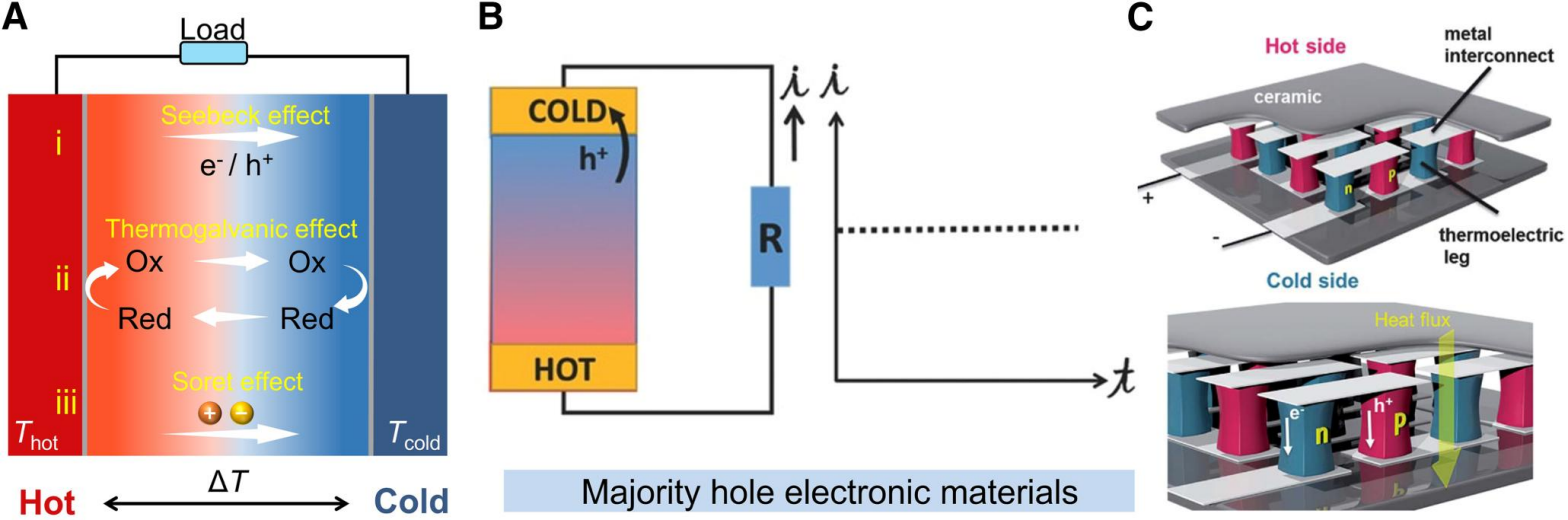
The working mechanism of electronic thermoelectric materials. (A) Schematic diagram of three thermoelectric effects under a temperature difference: (i) The Seebeck effect of electronic thermoelectric materials, (ii) the thermogalvanic effect of redox‐active electrolyte, and (iii) the Soret effect of non‐redox‐active electrolyte. (B) The majority hole electronic thermoelectric materials can generate a continuous output current.^30^ (Reprinted with permission. Copyright 2017, John Wiley and Sons). (C) Classical P–N TEG modules. The thermoelectric elements of N‐type and P‐type are connected in series by the metal interconnects and covered with ceramic plates.^32^ (Reprinted with permission. Copyright 2008, Royal Society Chemistry).

For a thermoelectric device located in a temperature field (Δ*T* = *T*_hot_ − *T*_cold_), there is a conversion from thermal energy to electrical energy, which is termed as thermoelectric effect (Figure 2A). The thermoelectric effect follows three main working mechanisms: Seebeck effect,^24^ thermogalvanic effect,^25^ and Soret effect.^26^ The Seebeck effect usually appears in electronic thermoelectric materials (e.g., conductors or semiconductors), while the thermogalvanic effect and the Soret effect can generate a voltage in ionic thermoelectric materials. According to the employed thermoelectric effect, thermoelectric devices can be divided into three categories: TEGs,^27^ TGCs,^28^ and TECs.^29^

### 3.1 Electronic thermoelectric materials

TEGs can generate stable and continuous electricity relying on the Seebeck effect, which is discovered in 1821 (Figure 2B).^30^ The Seebeck effect is a phenomenon that can produce a potential difference because the thermal energy induces the charge carriers' flow of electrical conductors or semiconductors. Generally, metallic materials show a rather low Seebeck coefficient, while semiconductors have better thermoelectric performance.^31^ For typical electronic thermoelectric materials, charge carriers are electrons or holes. Once applying the temperature difference (Δ*T*) across the electronic thermoelectric materials, more free charge carriers are induced on the hot side, leading to the charge carriers flowing from the hot side to the cold side because of entropy difference. Meanwhile, the accumulated carriers on the cold side tend to push the free‐charge carriers to flow back to the hot side. Consequently, the charge flow is balanced by the internal electrical field, forming a continuous potential difference (Δ*V*) under a temperature difference, which can be expressed as

(1) $$\Delta V=-S_{e}\Delta T,$$

here, *S*_*e*_ is the Seebeck coefficient, which is usually smaller than 100 μV K^−1^. The electronic thermoelectric materials can be classified into *p*‐type (*S*_*e*_ > 0, holes as carriers) and *n*‐type (*S*_*e*_ < 0, electrons as carriers). They can be connected in series (P–N, P–P, and N–N) and assemble arrays to improve the total outputs of voltage, current, and power (Figure 2C).^32^

### 3.2 Ionic thermoelectric materials

**FIGURE 3:**
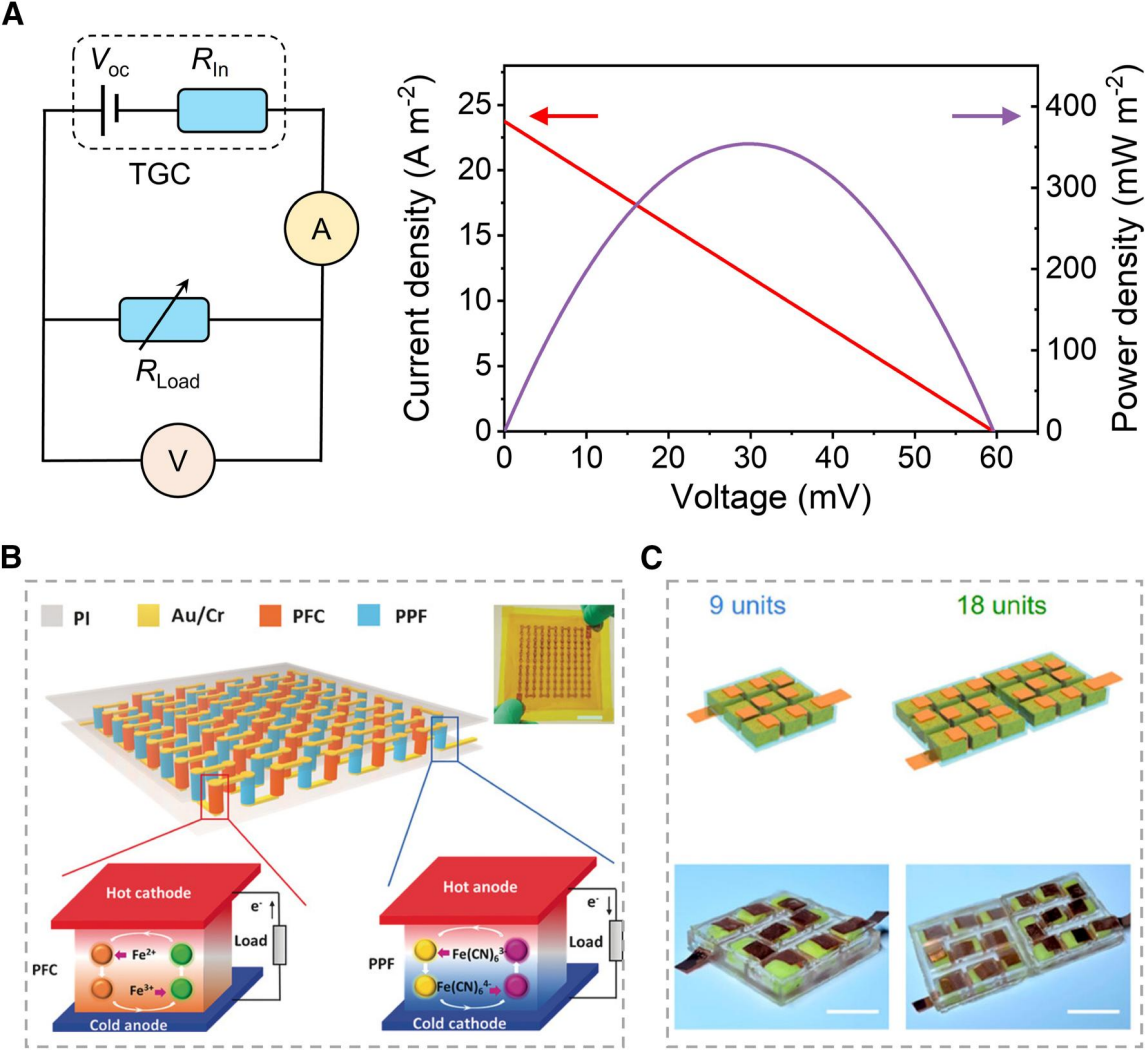
Working mechanisms of ionic thermoelectric materials of redox‐active electrolyte. (A) The equivalent electrical circuit of the TGCs that connect with an external load. (B) Typical P–N TGC modules.^36^ (Reprinted with permission. Copyright 2016, John Wiley and Sons). (C) Typical P–P TGC modules.^37^ (Reprinted with permission. Copyright 2022, American Chemical Society).

The recently developed ionic thermoelectric materials (TGCs and TECs) have relatively high thermopower (>1 mV K^−1^). In particular, polymer‐based quasi‐solid TGCs and TECs have intrinsic stretchability and flexibility, showing potential as portable power supplies. The TGC has a redox‐active electrolyte to generate continuous electricity via the thermogalvanic effect. When the Δ*T* is applied across the redox‐active electrolyte, a reversible redox reaction occurs at the hot and cold electrodes, which can be given as^33^:

(2) $${I_{\text{ox}}}^{m+}+\left(m-n\right)e^{-}⇌{I_{\text{red}}}^{n+},$$

where ${I_{\text{ox}}}^{m+}$ is the oxidizing ion, ${I_{\text{red}}}^{n+}$ is the reducing ion, and *m*–*n* is the mole quantities of the electrons transferred in the redox reaction. Thus, the redox couple ions will obtain or lose electrons at the hot and cold sides, generating a continuous potential difference (Δ*V*) between the two electrodes. The potential difference can be expressed as^34^:

$$\Delta V=\alpha \Delta T=\frac{s_{\text{red}}-s_{\text{ox}}}{nF}\Delta T=-S_{i}\Delta T,$$

where *F* is the Faraday constant and *s*_red_ and *s*_ox_ are the partial molar entropies of the I_red_ and I_ox_, respectively. Note that *α* is the temperature coefficient, whose sign is opposite to the sign of the thermopower of TGC, *S*_i_. The equivalent electrical circuit of the TGCs can be regarded as a power supply with an internal resistance *R*_In_ (Figure 3A).^35^ Similar to the electronic thermoelectric material, the *p*‐type (*S*_i_ > 0) and *n*‐type (*S*_i_ < 0) ionic thermoelectric materials also can be connected in series (P–N, P–P, and N–N) to compose the fundamental TGC element, which can be further assembled as TGCs to support the demands of output voltage, current, and power (Figure 3B,C).^36^, ^37^

**FIGURE 4:**
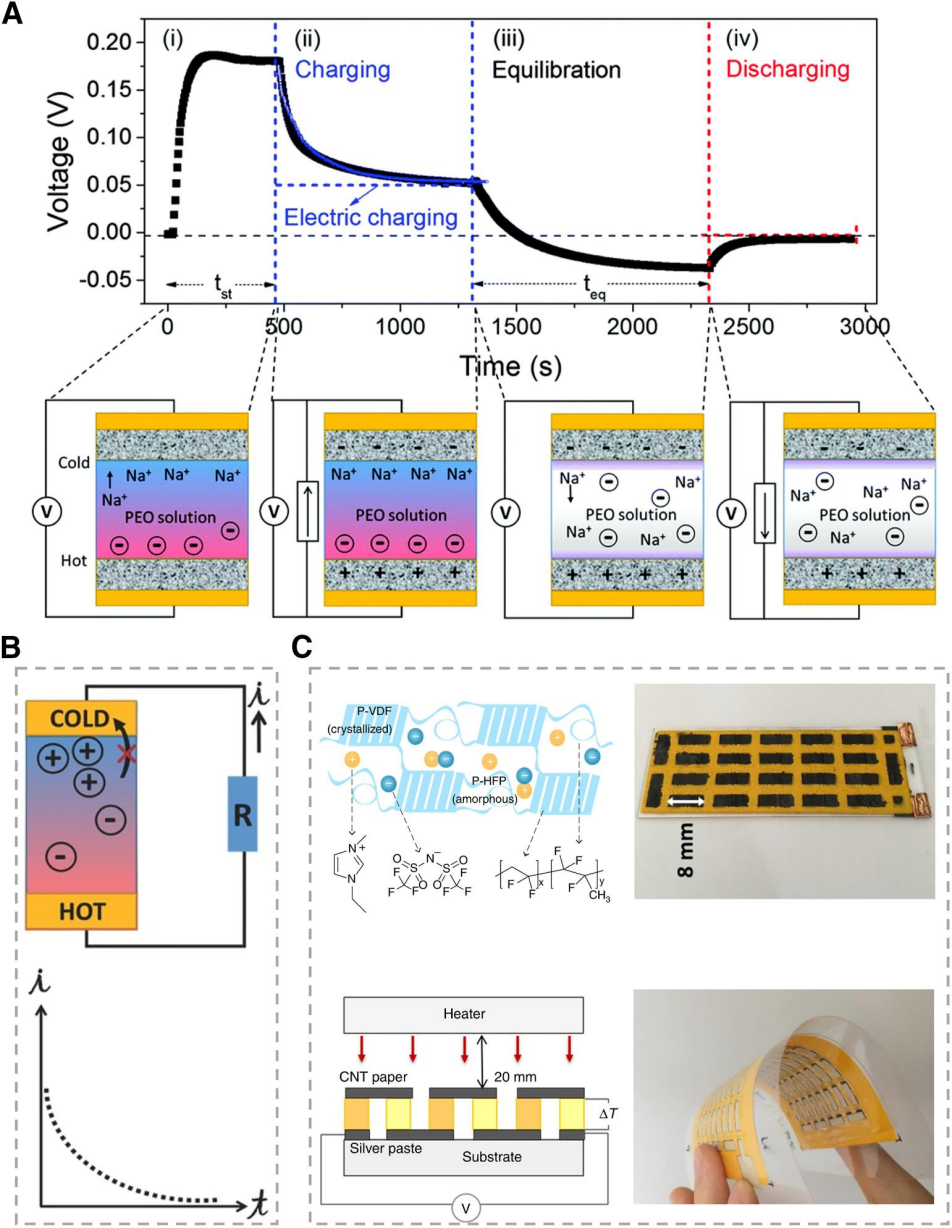
Working mechanisms of ionic thermoelectric materials with non‐redox‐active electrolytes. (A) The working principles of TEC. The transient potential difference can be used by an external load.^38^ (Reprinted with permission. Copyright 2008, Royal Society Chemistry). (B) The thermoelectric materials with non‐redox‐active electrolytes can generate an output current that decreases with time.^30^ (Reprinted with permission. Copyright 2017, John Wiley and Sons). (C) Typical P–N TEC modules.^40^ (Reprinted under the terms of the Creative Commons CC BY license. Copyright 2019, Springer Nature).

In addition to TGC, another kind of ionic thermoelectric material (TEC) with a non‐redox‐active electrolyte can generate a transient voltage (Figure 4A). When Δ*T* is applied across the non‐redox‐active electrolyte, the Soret effect promotes the diffusion of the ions from the hot to the cold side. Different ions accumulate at the interface between the different electrodes and the electrolyte, forming electric double layers (EDL) to provide transient current (Figure 4B).^38^ The thermodiffusion of the anions and cations is different, thus leading to the potential difference (Δ*V*), which can be expressed as^39^:

$$\Delta V=-S_{\text{td}}\Delta T=\frac{\left({\hat{S}}_{+}D_{+}\right)-\left({\hat{S}}_{-}D_{-}\right)}{e\left(D_{+}+D_{-}\right)},$$

where ${\hat{S}}_{+}$ and ${\hat{S}}_{-}$ are Eastman entropies of cations and anions, $D_{+}$ and $D_{-}$ are diffusion coefficients of cations and anions, and $S_{\mathrm{td}}$ is the thermopower of the TEC. The *n*‐type (*S*_td_ < 0) and *p*‐type (*S*_td_ > 0) TECs also can be connected in series (P–N, P–P, and N–N) to compose the fundamental TGC element, which can be further assembled as TECs to support the demands of output voltage, current, and power (Figure 4C).^40^

### 3.3 Thermoelectric materials for personalized healthcare

The thermoelectric materials for personalized healthcare should be flexible to conform to the human body and provide high output to driving biomedical devices. From the viewpoint of human conformal, electronic thermoelectric materials composed of semiconductors and metals show mechanical brittleness, and the ionic thermoelectric materials may face electrolyte leakage, which restrict the fabrication of flexible devices. In addition, the thermoelectric materials should provide high performance and peak output near human body temperature. However, most electronic thermoelectric materials only can reach high performance above 100°C, while poor near body temperature and the ionic thermoelectric materials will face the air instability of aqueous electrolytes.

Electronic thermoelectric materials can be divided into organic and inorganic categories. The inorganic materials should develop miniaturized thermoelectric legs to meet the requirement of processability and flexibility. Typical organic electronic thermoelectric materials, such as carbon nanotubes, graphene, polythiophene (PTh), polyaniline (PANi), poly(3,4‐ethylenedioxythiophene) (PEDOT), polypyrrole (PPy), and their chemical doping composites, are promising candidates for personalized healthcare. On the one hand, the organic electronic thermoelectric materials have developed a power factor higher than 100 μW m^−1^ K^−2^ near room temperature. On the other hand, the composite of organic electronic thermoelectric materials and stretchable elastic insulators can contribute to improving the flexibility and processability of the materials.^41^

Aqueous TGCs and TECs have a risk of electrolyte leakage, while the polymer‐based quasi‐solid TGCs and TECs enable preserving the electrolyte by the polymer networks to avoid such risk, which can be used as portable power supplies for personalized healthcare. The typical TEG contains a polymer matrix, redox couple electrolytes, and electrodes. The redox couple electrolytes, such as Fe^3+^/Fe^2+^, Fe(CN)_6_^3−^/Fe(CN)_6_^4−^, I_3_^−^/I^−^, Np^4+^/Np^3+^, and Pu^4+^/Pu^3+^, are usually dispersed in environmentally friendly water. The electrodes, such as platinum, carbon, conducting polymers, and polymer matrix, are further integrated with the electrolyte. To improve the output power, electrodes with controlled morphology can be introduced. The typical TGC contains a polymer matrix, non‐redox‐active electrolyte, and electrodes. The polymer matrix can choose stretchable and flexible nonaqueous ionogels or hydrogels. The thermopower of current TGC achieves −15 mV K^−1^ for N‐type and +17 mV K^−1^ for P‐type in nonaqueous ionogels.^42^ For hydrogels, the thermopower could even be −37 mV K^−1^ (N‐type).^26^

## 4 BIOMEDICAL APPLICATIONS

After being effectively integrated with biomedical devices, thermoelectrics can be applied in wearable and implantable applications for monitoring and diagnosis. Compared with the previous power supplies, thermoelectric energy harvesting has advantages in continuous power.

### 4.1 Wearable applications

Long‐term and accurate vital signal monitoring (pulse rate, blood pressure, and body temperature) is helpful to provide a thorough evaluation of the physiological state of patients. The human body's skin has a constant temperature of ∼37°C, which can be employed as a stable heat source in wearable applications. Thermoelectrics can harvest body heat into electricity via the thermoelectric effect, producing continuous and stable energy output for powering wearable biomedical sensors at any time.

**FIGURE 5:**
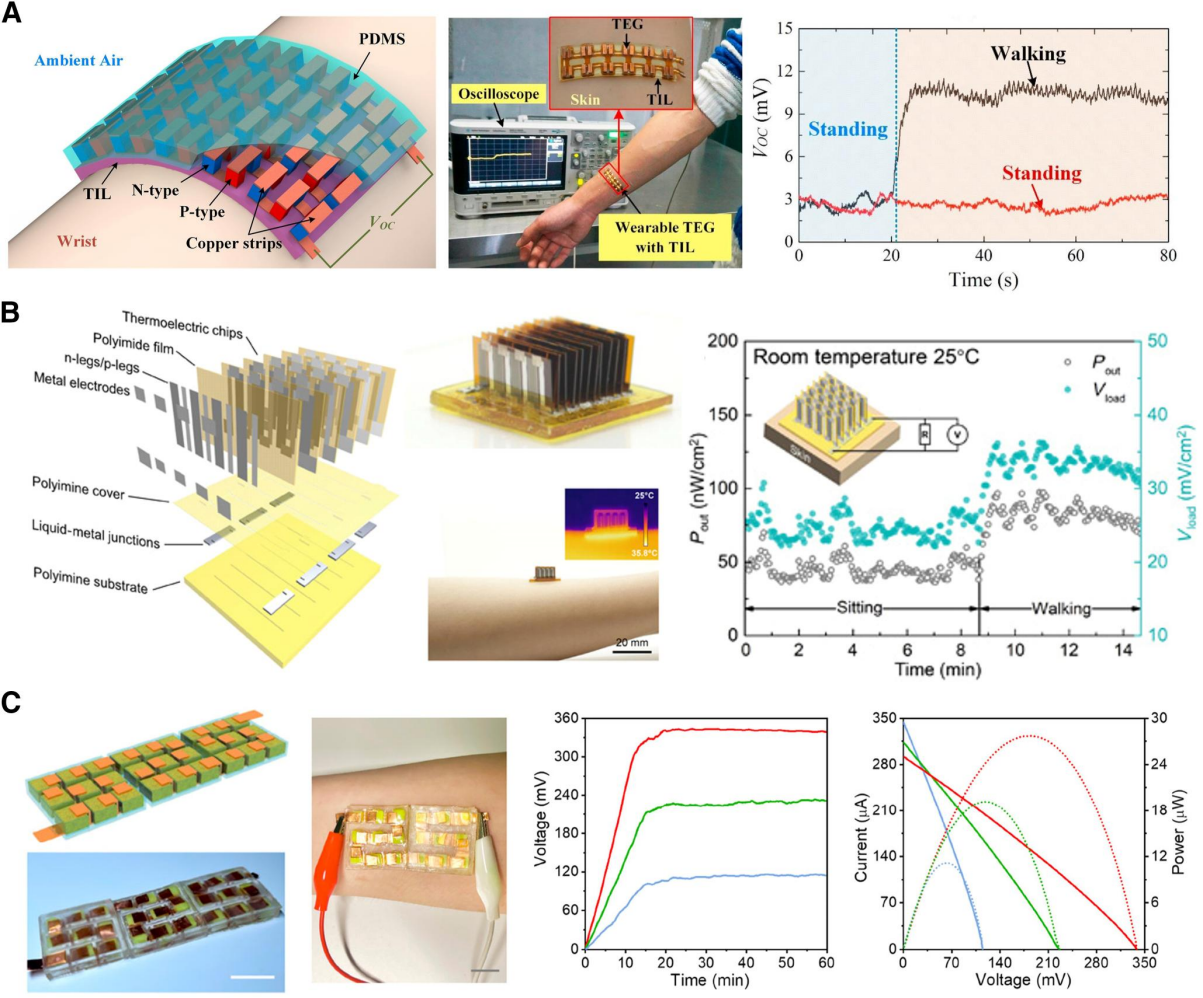
Wearable applications of continuous TEG and TGC. (A) A TEG is composed of bulk N‐type and P‐type legs.^45^ (Reprinted with permission. Copyright 2017, Elsevier). (B) A TEG based on 2D thermoelectric chips that are composed of N‐type (Bi_2_Te_2.8_Se_0.3_) and P‐type (Bi_0.5_Sb_1.5_Te_3_) materials.^46^ (Reprinted under the terms of the Creative Commons Attribution‐NonCommercial license. Copyright 2020, American Association for the Advancement of Science). (C) TGC with 27 P‐type units.^37^ (Reprinted with permission. Copyright 2022, American Chemical Society).

The TEGs have been widely explored in wearable applications because the inorganic semiconductors and conductors have advantages in long‐term stable operations. For wearable applications, TEGs require not only flexibility to fit undulated and dynamic human skin but also high integration density of the fundamental elements to improve the output power. Combining the two‐dimensional (2D) or three‐dimensional (3D) thermoelectric legs with the flexible substrates could fabricate flexible TEGs.^43^, ^44^ The TEG achieves an output voltage of 3 mV without motion and 11.2 mV with walking when people wear it at the ambient temperature of 19°C (Figure 5A).^45^ Compared with the bulk 3D legs, flexible TEG based on 2D thermoelectric legs can integrate more fundamental elements in the same heat source areas. Figure 5B shows a TEG based on 2D thermoelectric chips.^46^ Here, N‐type (Bi_2_Te_2.8_Se_0.3_) and P‐type (Bi_0.5_Sb_1.5_Te_3_) materials were thermally deposited onto flexible polyimide films and completed P–N connection in series via metal electrodes, serving as 2D thermoelectric chips. Stretchable and self‐healable polyimine served as the thermal interface layer, which was further assembled with the 2D thermoelectric chips and liquid metal electrodes. When attached to a forearm at the ambient temperature (25°C), this TEG can produce an output voltage of 25 mV cm^−2^ and a power density of 45 nW cm^−2^ in the sitting state and that of 33 mV cm^−2^ and 83 nW cm^−2^ in the walking state.

**FIGURE 6:**
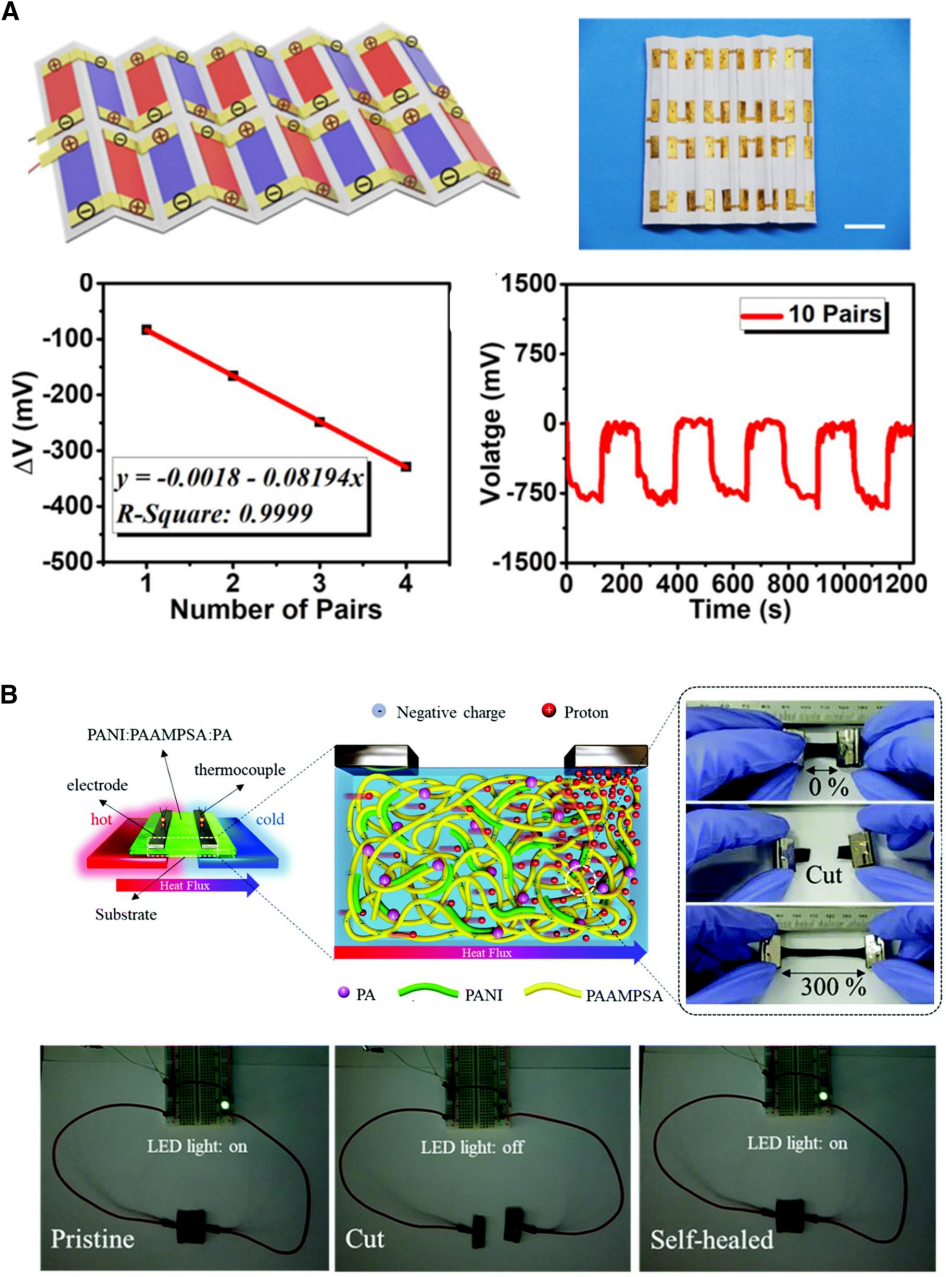
Wearable applications of thermoelectric capacitor (TEC). (A) The TECs with 10 P–N units in series.^48^ (Reprinted with permission. Copyright 2022, American Chemical Society). (B) Self‐healing TEC based on the physically cross‐linked networks.^49^ (Reprinted with permission. Copyright 2020, Royal Society Chemistry).

The TGCs and TECs based on ionic thermoelectric materials can use cheap electrode, electrolyte, and polymeric matrix materials to achieve Seebeck coefficients hundreds of times higher than TEGs. The TGC is usually a structure with two electrodes sandwiching the electrolyte. The TEC can be constructed into a planar structure in addition to the sandwiched structure. Although both TEGs and TGCs can be used for wearable applications, there are differences in their real operation. The TGCs can continuously convert body heat to electricity at a stable temperature difference, while TGCs work in a discontinuous mode, which needs human intervention to connect to the external load once worn on the human body and then reconnect after balancing the temperature difference.^47^ Figure 5C shows assembled thermocell arrays connected with P‐type units in series. The TGCs with 27 P‐type units achieve a maximum output power of 28 μW and a total voltage of 342 mV at the Δ*T* of 10 K.^37^ When TECs are connected with P–N types in series, 10 P–N units achieve a voltage of 823.3 mV (Figure 6A).^48^ In addition, The TGC enables self‐healing properties due to physically cross‐linked networks, overcoming the limitation of durability for practical applications (Figure 6B).^49^

### 4.2 Implantable applications

**FIGURE 7:**
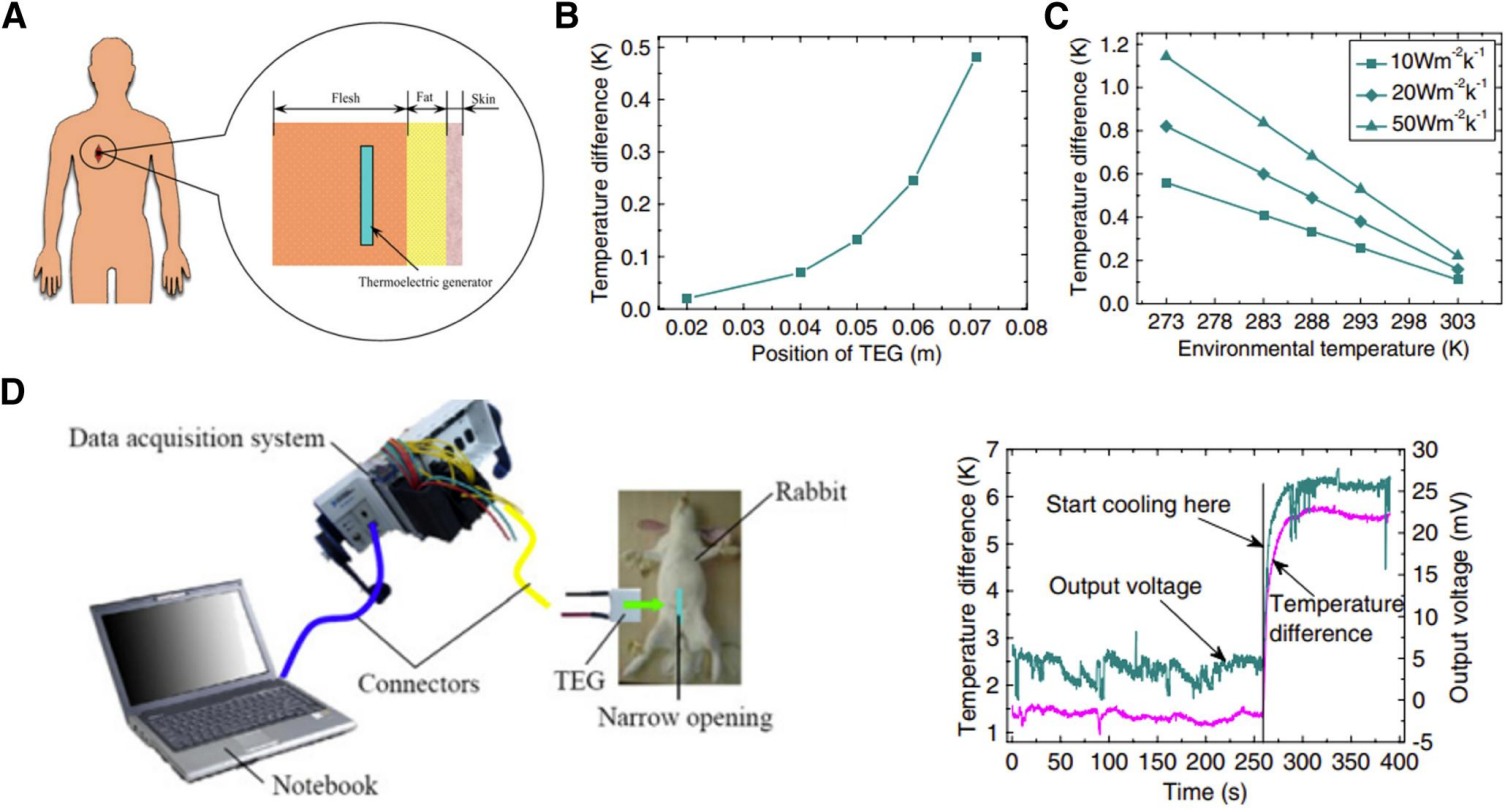
Implantable applications of themoelectrics.^51^ (A) Schematic diagram of the implantable location in the human body. (B) The thermoelectric temperature difference in different implantable positions. (C) Effect of skin convection on the thermoelectric temperature difference. (D) The output voltage after implanting the thermoelectric device into a rabbit. (Reprinted with permission. Copyright 2007, IOP Publishing).

Biomedical implantable devices, like cardiac defibrillators, cochlear implants, cardiac pacemakers, and neurological stimulators, have been involved in the safeguarding of human life. However, the lifespan of the implantable devices is limited by the batteries, which require to be replaced with surgery about every 10 years. Although human skin can be considered constant temperature, the muscle layer exists a temperature gradient between the body core and the skin surface during different physical activities (Figure 7A). Thus, the TEGs and TGCs are all promising candidates for continuous power supply for implantable devices.

The first concern is the embedded position where thermoelectric devices enable obtaining a maximum Δ*T*. According to the Pennes bio‐heat equation,^50^ a one‐dimensional tissue model can be used to calculate the temperature gradient, which can be expressed as

$$\rho c\frac{\partial T}{\partial t}=k\frac{\partial^{2}T}{\partial x^{2}}+Q_{b}+Q_{m}.$$

where *c* and *ρ* are the heat capacity and tissue density, respectively, *T* is the tissue temperature, *k* is the tissue thermal conductivity, and *Q*_*b*_ and *Q*_*m*_ are the heat flux of blood and metabolic, respectively. If a thermoelectric device with the size of 20 × 20 × 3 mm is implanted into the human body, the calculation result shows a maximum temperature difference of 0.482 K at the position near the body core of 0.071 m (Figure 7B).^51^ It means that implanted thermoelectric devices should be embedded as close to the skin layer as possible to ensure safe and high‐efficiency operation. Besides, the temperature difference can be further increased up to 1.2 K when the convection coefficient is increased to 50 W m^−2^ K^−1^ and the environmental temperature is decreased to 273 K (Figure 7C). After being implanted into a rabbit, the commercial thermoelectric generator has a temperature difference of 1.3 K and generates an output voltage of about 5 mV (Figure 7D).

The other concern is the biocompatibility and mechanical properties of the implanted thermoelectric devices.^21^ On the one hand, implantable thermoelectric devices exposed to the human physiological environment potentially lead to allergies and rejection, which may cause pain and even threaten the life of patients. Thus, a biocompatible encapsulation layer is required to insulate the human tissue and thermoelectric devices. On the other hand, better mechanical properties of thermoelectric devices with better mechanical properties should be developed to avoid failing to work as affected by strenuous human activity.

## 5 CONCLUSIONS AND PERSPECTIVES

The thermoelectric technology that enables continuous harvesting of the heat energy dissipated from the human body holds the potential to satisfy the long‐term energy consumption of the miniaturized and decentralized personalized healthcare platforms. In this review, we have thoroughly summarized thermoelectric energy harvesting for personalized healthcare, covering the research from the fundamental concepts of the different thermoelectrics to the wearable and implantable applications. Although thermoelectric energy harvesting of body heat for decentralized biomedical platforms has experienced rapid development in theory and technologies in the last decades, there remain challenges to improve the energy conversion efficiency and help push the technique into commercial applications. Herein, we will discuss the most prominent research directions for designing next‐generation thermoelectric devices on three significant aspects: (1) high‐performance thermoelectric devices, (2) new configuration thermoelectric devices, and (3) commercialization of thermoelectric devices.

1. High‐performance thermoelectric devices

The high‐performance thermoelectric device enables high power output in a small size, which is beneficial to further applying the thermoelectric devices in various scenarios. Two approaches can be considered. On the one hand, the single thermoelectric elements should be improved, thus achieving high output voltage and power of the integrated module. For electronic thermoelectric materials, the thermoelectric figure of merit (ZT) can be improved through the rational design of band engineering and structural optimization.^52^, ^53^ For the ionic thermoelectric materials, the thermopower and power density can be improved by increasing entropy difference,^54^ ion–ion interactions,^42^ and ionic conductivity.^47^ On the other hand, it is also important to increase the temperature difference of the thermoelectric devices through the heat exchange design.^55^ The thermally conductive materials can be introduced to enhance the heat exchange between the heat source and the hot side of the thermoelectric device. The heat sink can be integrated with the thermoelectric device to decrease the temperature of the cold side.^56^

2 New configuration designs

To extend the application scope, new configuration designs of thermoelectric devices are demanded. For wearable applications, most current thermoelectric devices are based on integrating the fundamental elements into blocks. To meet aesthetics and everyday comfort, thermoelectric devices can be structured as textiles.^57^, ^58^ The textiles are composed of highly integrated thermoelectric fibers, whose output power and voltage are related to the quantities of thermoelectric elements.^59^ Incorporating thermoelectric textiles on the human body is promising to achieve maximum thermal energy harvest for remote personalized healthcare with minimal intervention. In addition, according to the applicable environment, thermoelectric devices also can configure specific functions. For example, thermoelectric devices can integrate with flexible radiative coolers to achieve lower cold side temperatures during outdoor activities or develop freeze‐tolerant functions to be applied in extremely cold environments.^60^ For implantable applications, different stacking ways of thermoelectric devices in the human body should be explored to ensure enough output power as well as minimal implantable sensation. Meanwhile, the temperature difference of the thermoelectric device inside the human body is much lower than that outside the human body. Thus, biocompatible encapsulation layers need to be developed to enhance the thermal conduction between the hot side and the in vivo physiological environment and inhibit the heat exchange between the cold side and the internal environment.^61^

3 Commercialization of thermoelectric devices

Both clinical safety and economic benefits should be taken into consideration before the commercialization of thermoelectric devices into marketable biomedical products. The certifications of wearable thermoelectric devices are easier than implantable thermoelectrics because the evaluation of the product in a long‐term physiological environment is time and cost‐consuming. The government, commercial institutions, and research institutions should cooperate to promote laboratory research in the biomedical market. In addition, customized thermoelectrics are needed in specific cases, which can combine computational inverse design techniques and machine learning to improve design efficiency.^62^

In summary, thermoelectric energy harvesting from body heat shows great potential to provide sustainable electricity for wearable and implantable biomedical platforms, which is a crucial prerequisite to supporting remote and personalized healthcare. However, there remains a lot of room for improvement in their conversion efficiency. Besides, it is necessary to develop encapsulation and integration techniques to improve the total outputs of voltage and power. Furthermore, biosafety standards need to be developed for implantable applications. With the growing research in thermoelectric materials and devices, we believe they will change the landscape of personalized healthcare in the future.

## AUTHOR CONTRIBUTIONS

Wei Gao, and Feili Lai conceived the idea and designed the review. All the authors wrote, discussed and commented the review.

## CONFLICT OF INTEREST

The authors declare that they have no conflict of interest.
